# Negative Regulation of Syntaxin4/SNAP-23/VAMP2-Mediated Membrane Fusion by Munc18c *In Vitro*

**DOI:** 10.1371/journal.pone.0004074

**Published:** 2008-12-31

**Authors:** Fiona M. Brandie, Veronica Aran, Avani Verma, James A. McNew, Nia J. Bryant, Gwyn W. Gould

**Affiliations:** 1 Henry Wellcome Laboratory of Cell Biology, Division of Biochemistry and Molecular Biology, Institute of Biomedical and Life Sciences, University of Glasgow, Glasgow, United States of America; 2 Department of Biochemistry and Cell Biology, Rice University, Houston, Texas, United States of America; Thomas Jefferson University, Kimmel Cancer Center, United States of America

## Abstract

**Background:**

Translocation of the facilitative glucose transporter GLUT4 from an intracellular store to the plasma membrane is responsible for the increased rate of glucose transport into fat and muscle cells in response to insulin. This represents a specialised form of regulated membrane trafficking. Intracellular membrane traffic is subject to multiple levels of regulation by conserved families of proteins in all eukaryotic cells. Notably, all intracellular fusion events require SNARE proteins and Sec1p/Munc18 family members. Fusion of GLUT4-containing vesicles with the plasma membrane of insulin-sensitive cells involves the SM protein Munc18c, and is regulated by the formation of syntaxin 4/SNAP23/VAMP2 SNARE complexes.

**Methodology/Principal Findings:**

Here we have used biochemical approaches to characterise the interaction(s) of Munc18c with its cognate SNARE proteins and to examine the role of Munc18c in regulating liposome fusion catalysed by syntaxin 4/SNAP23/VAMP2 SNARE complex formation. We demonstrate that Munc18c makes contacts with both t- and v-SNARE proteins of this complex, and directly inhibits bilayer fusion mediated by the syntaxin 4/SNAP23/VAMP2 SNARE complex.

**Conclusion/Significance:**

Our reductionist approach has enabled us to ascertain a direct inhibitory role for Munc18c in regulating membrane fusion mediated by syntaxin 4/SNAP23/VAMP2 SNARE complex formation. It is important to note that two different SM proteins have recently been shown to stimulate liposome fusion mediated by their cognate SNARE complexes. Given the structural similarities between SM proteins, it seems unlikely that different members of this family perform opposing regulatory functions. Hence, our findings indicate that Munc18c requires a further level of regulation in order to stimulate SNARE-mediated membrane fusion.

## Introduction

Studies in a variety of eukaryotic systems have led to the identification of a family of proteins that function in membrane fusion using a mechanism highly conserved through evolution [1]. In its simplest form, the SNARE hypothesis states that the fusion of a donor membrane with its target is mediated by the specific pairing of target (t)-SNAREs with a cognate vesicle (v)-SNARE [1]. Specific v- and t-SNARE combinations are involved in all membrane trafficking events in eukaryotic cells [1]. Multiple lines of evidence indicate that SNARE proteins constitute the minimal machinery required for bilayer fusion [1]. Importantly, reconstitution of t-SNARE and v-SNARE proteins in synthetic donor and acceptor liposomes drives fusion of the two populations [2]. Although the use of this *in vitro* fusion assay clearly indicates that the formation of specific SNARE complexes is sufficient to catalyse membrane fusion, this fusion occurs at a rate far slower than that observed in physiological systems [2]. Biochemical and genetic approaches have implicated other proteins as being required for the regulation of SNARE complex formation. One such family is the Sec1p/Munc18 (SM) proteins [3].

Like the SNAREs, SM proteins are highly conserved through evolution, and understanding their precise role in membrane fusion represents an important question in cell biology [3]. Munc18a was originally identified as a Syntaxin1A (Sx1A)-binding protein whose binding to Sx1A precludes SNARE complex formation [4], [5]. Consistent with this, crystallographic studies have revealed that Munc18a is an arch shaped molecule that cradles monomeric Sx1A in a closed conformation that is incompatible with the entry of Sx1A into SNARE complexes [6]. These data support a model in which SM proteins hold their cognate syntaxins in a closed conformation and thus regulate SNARE complex assembly, perhaps by facilitating the switch of syntaxins from their closed to a more open conformation [7]. The Munc18a/Sx1A crystal structure reveals contacts between the inner arch of Munc18a and almost the entire length of Sx1As cytosolic domain [6].

In striking contrast to the interaction between Munc18a and Sx1A, the extreme N-terminal region of other syntaxins are both necessary and sufficient to capture their cognate SM proteins. For example, the N-terminal 44 residues of Sed5p are sufficient to bind the SM protein Sly1p [8], [9]. The crystal structure of this interaction reveals that the N-terminal peptide of the syntaxin inserts into a hydrophobic pocket on the outer face of the SM protein [9]. This interaction is consistent with the SM protein binding to either a closed or open conformation of the syntaxin. The finding that different SM proteins bind their cognate syntaxins via strikingly different mechanisms has severely hampered formulation of a unifying hypothesis describing the mechanism(s) by which SM proteins regulate SNARE-mediated membrane fusion. We have previously demonstrated that the SM protein Vps45p uses two distinct modes of binding to interact with its cognate SNARE proteins throughout the SNARE complex assembly/disassembly cycle [10], [11]. Vps45p dissociates from its monomeric syntaxin prior to formation of *trans*-SNARE complexes, and then re-associates following membrane fusion and the conversion of *trans*-SNARE complexes to *cis*-SNARE complexes [10], [11]. We suggest that all syntaxin/SM pairs interact using *both* of these modes of binding at different stages of the SNARE assembly/disassembly cycle. This model would allow the SM protein to prevent futile reformation of SNARE complexes following the action of the ATPase NSF, and allow the SNARE proteins to recycle for further rounds of membrane fusion. Since different syntaxin/SM pairs are likely subject to different levels of regulation, the rate-limiting step in the SNARE assembly/disassembly cycle may differ for different membrane trafficking pathways. Thus, in the case of Sx1A/Munc18a the SNARE complex controls a tightly regulated fusion pathway (regulated exocytosis), and Munc18a binds preferentially to Sx1A in the closed conformation with high affinity [7]. In contrast, Sed5p/Sly1p is involved in constitutive trafficking pathways, and is likely regulated in a distinct manner. The binding seen *in vitro* possibly reflects the highest affinity interaction, with the lower affinity interactions overlooked.

This may be exemplified by Munc18a that cradles monomeric Sx1A in its closed conformation, as shown by the crystal structure described above [6]. Mutant versions of Sx1A that are unable to adopt the closed conformation are unable to bind Munc18a in this manner [7]. However, it has recently been demonstrated that Munc18a also binds to the assembled Sx1A-SNAP25-VAMP complex in which Sx1A is in a more open conformation [12], and also the N-terminal peptide of Sx1A in manner similar to that observed for Munc18c and Sly1p binding to Sx4 and Sed5p respectively [13], [14]. Similarly, Sec1p, which was originally thought to bind exclusively to assembled SNARE complexes [15], has recently been shown to bind to unassembled t-SNAREs [16]. These observations underscore the need to accommodate multiple modes of interaction in any model describing SM protein function.

The insulin-dependent delivery of GLUT4-containing vesicles to the plasma membrane is a specialised example of regulated membrane trafficking [17]. GLUT4-containing vesicles are mobilised to the cell surface in response to insulin binding its receptor, where they dock and fuse [17]. Studies in both fat and muscle cells have established that GLUT4-vesicle fusion is mediated by the SM protein Munc18c which binds with high affinity to the plasma membrane t-SNARE Sx4 [18]. Sx4 along with SNAP-23 forms a functional SNARE complex with the v-SNARE VAMP2, carried by GLUT4-containing vesicles [18]. Numerous studies have established the importance of these proteins in GLUT4 translocation [17], [19]. Homozygous knockout of Munc18c in mice results in an increased sensitivity of GLUT4 exocytosis in response to insulin, suggesting that Munc18c negatively regulates GLUT4 exocytosis [20]. In addition, over-production of Munc18c in 3T3-L1 adipocytes has been shown to inhibit insulin-stimulated GLUT4 translocation [21]–[23]. By contrast, Munc18c heterozygous knockout mice exhibit impaired insulin sensitivity [24].

We set out to define the role of Munc18c in membrane fusion driven by Sx4/SNAP23/VAMP2. The *in vitro* liposome fusion assay offers a powerful tool with which to finely dissect the mechanistic basis of SM protein function [12], [16]. Here we use this assay, catalysed by Sx4/SNAP23/VAMP2, to investigate the role of Munc18c in bilayer fusion.

## Results and Discussion

### Munc18c exhibits dose-dependent inhibition of Sx4-mediated liposome fusion

**Figure 1:**
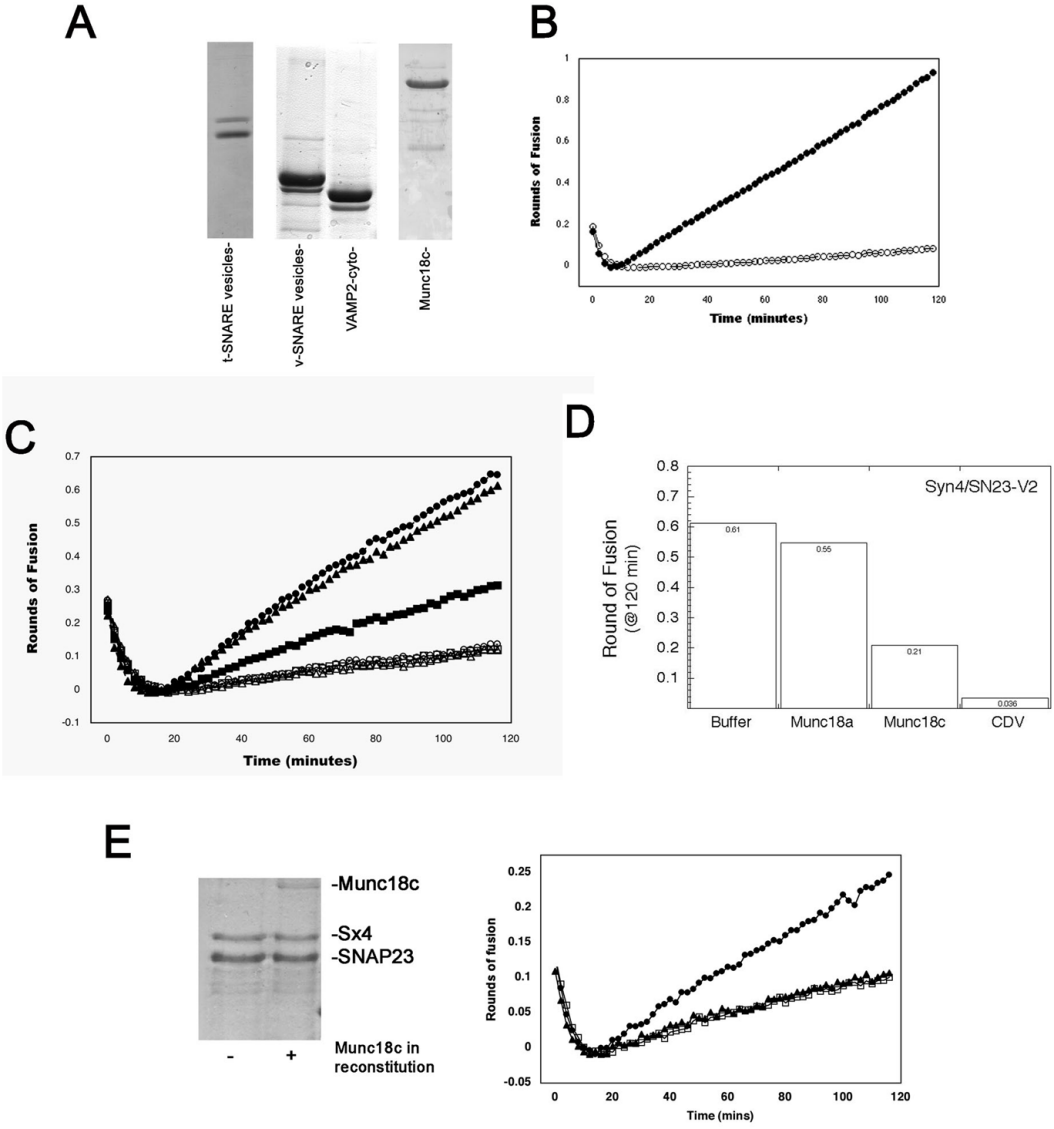
Munc18c inhibits Syntaxin 4/SNAP-23/VAMP2-mediated bilayer fusion *in vitro*. *(A,B) VAMP2 and syntaxin 4/SNAP23 form a functional complex capable of fusing liposomes.* (A) Coomassie blue stained gels of the recombinant proteins used in this study. Shown are samples of the t- and v- proteoliposomes used for fusion assay (10 µl each), the cytoplasmic domain of VAMP2 (VAMP2-cyto, 5 µg) and 5 µg of purified Munc18c were separated by SDS-PAGE and stained with Coomassie Blue. (B) Fluorescently labelled donor VAMP2 liposomes, 5 µl, were pre-incubated at 4°C overnight with 45 µl of unlabelled acceptor syntaxin 4/SNAP23 liposomes (with the addition of 2 µl of A200 to correct for volume) and fusion between the two vesicle populations monitored by measuring NBD fluorescence at 520 nm every 2 min (filled circles). As a control fluorescence was monitored with the addition of 2 µl of VAMP2-cyto (open circles). The fluorescence was converted into rounds of fusion as outlined in [16]. Data shown is representative of over 6 experiments of this type. *(C) Munc18c added to v-SNARE and t-SNARE vesicles inhibits fusion in a dose dependant manner*. Munc18c, dialysed against glycerol free A200 buffer, was added directly to the fusion assay set up as outlined in panel A and the mixture incubated at 4°C overnight. Fusion between the two vesicle populations in the absence (filled circles) and presence of Munc18c at two different ratios to syntaxin4/SNAP23 (2∶1, filled triangles, 10∶1, filled squares) was monitored by measuring NBD fluorescence at 520 nm every 2 min. As a control, fluorescence was monitored in each population in the presence of 2 µl of VAMP2-cyto (open circles, open triangles and open squares respectively). The fluorescence was converted into rounds of fusion as outlined [16]. The experiment shown was repeated four times with quantitatively similar results. *(D) Munc18a does not inhibit fusion*. Unlabeled acceptor liposomes containing the syntaxin4/SNAP23 t-SNARE complex (20 µl) were mixed with labelled VAMP2 liposomes (2.8 µl) in a total reaction volume of 70 µl with or without Munc18a or Munc18c. The final concentration of t-SNAREs in the reaction was 3.7 µM protein and 1.6 µM VAMP2. Munc18a or Munc18c were added at 10 µM. SNARE liposome were preincubated with SM proteins for 3 h at 4°C prior to fusion at 37°C and fusion assayed as in *B*. *(E) Munc18c pre-incubated with t-SNAREs prior to reconstitution inhibits fusion facilitated by VAMP2 and syntaxin 4/SNAP23*. Equimolar amounts of Munc18c, dialysed against A200 buffer, and t-SNARE complex were mixed overnight at 4°C. As a control the same amount of t-SNARE was mixed overnight at 4°C with A200 buffer alone. OG was added to maintain the concentration at 1%. Reconstitution was then carried out as described and aliquots (10 µl) of t-SNARE vesicles analyzed by SDS-PAGE and Coomassie staining (left panel) the left hand panel. Fusion was monitored between vesicles containing t-SNAREs alone (filled circles) and t-SNAREs premixed with Munc18c (filled triangles) with v-SNARE vesicles. Data from a representative experiment is shown (right hand panel), repeated three times with quantitatively similar data. As a control, fusion was also monitored between vesicles containing t-SNAREs alone and v-SNARE vesicles in the presence of excess VAMP2-cyto (open squares). N.B. Lower quantities of input SNAREs were employed due to dilution of t-SNAREs with Munc18c during the reconstitution, hence the fusion rates in this experiment are lower than those in B and C. Note that the addition of Munc18c reduced fusion by the same amount as VAMP2-cyto in these experiments.

Two different SM proteins have recently been shown to stimulate SNARE-mediated liposome fusion [12], [16]. To assess whether this phenomenon is widely conserved, we examined the effect of the SM protein Munc18c on the liposome fusion assay catalysed by Sx4/SNAP23/VAMP2 SNARE complex formation. Fig. 1A shows the recombinant proteins used in this experiment.

Bilayer fusion catalysed by this SNARE complex was found to exhibit similar rates of fusion to that reported for the neuronal SNARE complex Sx1A/SNAP25/VAMP and the yeast exocytic SNARE complex Sso1p/Sec9p/Snc2p (Fig 1B) [12], [16]. Rates of fusion catalysed by Sx1A/SNAP25/VAMP and Sso1p/Sec9p/Snc2p can be significantly enhanced by the addition of their cognate SM proteins, Munc18a and Sec1p, respectively [12], [16]. To investigate the role of Munc18c on Sx4/SNAP23/VAMP2 mediated fusion, we added purified recombinant Munc18c to the liposome fusion assay presented in Fig 1B. Addition of Munc18c at molar ratios of either 2∶1 or 10∶1 Munc18c:t-SNARE complexes inhibited liposome fusion catalysed by this SNARE complex in a dose-dependent manner (Fig 1C). Importantly, addition of an equivalent amount of Munc18a, an SM protein that does not bind to Sx4, had no effect on fusion (Fig. 1D). To further validate this result, equimolar amounts of Munc18c and t-SNAREs were incubated prior to reconstitution into acceptor liposomes. Unbound Munc18c was removed by floatation of liposomes prior to assay. Fig 1E shows that pre-binding of Munc18c to Sx4/SNAP23 binary complexes in this manner completely abolished fusion.

### Munc18c binds the v-SNARE VAMP2

Although most research on SM protein function has focussed on their interaction with their cognate syntaxins, it is becoming apparent that interactions with non-syntaxin SNAREs also plays an important role in a conserved feature of SM protein function. We have previously shown that the yeast SM protein Vps45p not only binds directly to its cognate syntaxin, Tlg2p, but also to the assembled SNARE complex and also the v-SNARE Snc2p [11]. Similarly, in addition to binding Sed5p and the assembled SNARE complex, Sly1p also binds directly to the non-syntaxin SNAREs Bet1p, Bos1p, Sft1p and Gos1p [25]. Furthermore, in addition to its high affinity interaction with monomeric Sx1A, Munc18a also binds to the assembled Sx1A/SNAP25/VAMP2 SNARE complex [12] and VAMP2 alone [26].

**Figure 2:**
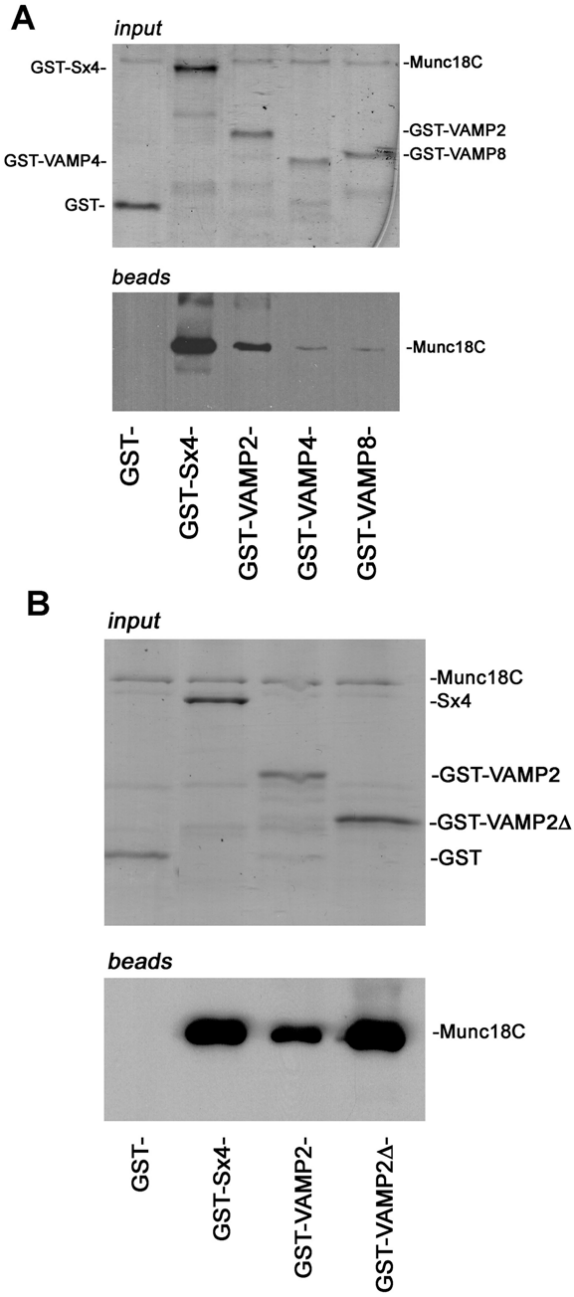
Munc18c binds directly to the cytosolic domains of Syntaxin 4 and VAMP2. (A) 5 µg of either GST, or GST fused to the cytosolic domain of Sx4, VAMP2, VAMP 4 or VAMP 8, immobilised on glutathione Sepharose were incubated overnight at 4 C with 5 µg His-tagged Munc18c in a volume of 500 µl. After extensive washing in binding buffer, SDS-PAGE and immunoblot analysis was used to determine which of the GST proteins Munc18c had bound to. Upper panel represents a Coomassie stained gel of input proteins; lower panel shows an immunoblot for bound Munc18c (B) The ability of Munc18c to bind to a version of GST-VAMP2 harbouring only the SNARE motif of the v-SNARE was assessed as in (A). Data are representative of four experiments of this type.

Munc18c has been reported to bind directly to monomeric Sx4, and also to the assembled Sx4/SNAP23/VAMP2 SNARE complex [27]–[29]. Fig 2 demonstrates that Munc18c also binds directly to the SNARE motif of the v-SNARE VAMP2. Purified recombinant Munc18c binds to GST-fusion proteins harbouring the cytosolic domains of Sx4 and VAMP2, but not those of the unrelated v-SNAREs VAMP4 and VAMP8 (Fig 2A). Quantification revealed that Munc18c binding to VAMP4 or VAMP8 was consistently less than 14% of that observed for VAMP2. The interaction of other SM proteins with v-SNAREs is mediated through the SNARE motif [11], [25]. Similarly, Fig 2B shows that Munc18c binds directly to the SNARE motif of VAMP2.

**Figure 3:**
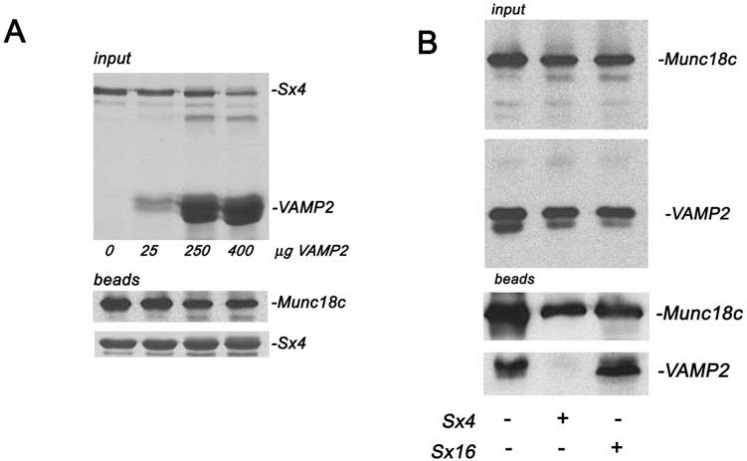
Syntaxin 4 disrupts the interaction of Munc18c with VAMP2. (A) 5 µg of M18c was immobilised on Ni-NTA agarose and incubated overnight with an excess (10 µg) of Sx4 cytosolic domain (residues 1–273) (final concentration 0.7 µM Munc18c, 3.3 µM Sx4 in the initial incubation). After extensive washing, the pre-formed complex was subsequently incubated with increasing concentrations of VAMP2 cytosolic domain (residues 1–92) as indicated. Upper panel shows a Coomassie stained gel of the input proteins; lower panel shows an immunoblot of the beads probed with anti-Munc18c, or stained with Coomassie to show levels of Sx4. (B) 5 µg of M18c was immobilised on Ni-NTA agarose and incubated overnight with an excess (10 µg) of VAMP2 cytosolic domain (final concentration 0.7 µM Munc18c, 7 µM VAMP2 in the initial incubation). The upper panel (input) shows an immunoblot analysis of these beads after extensive washing confirming the presence of both Munc18c and VAMP2. This preformed complex was then incubated alone or with 250 µg of Sx4 cytosolic domain or (as a control) 250 µg Sx16 cytosolic domain (residues 1–269) (both t-SNAREs at a final concentration 16.7 µM), as indicated. Immunoblot analysis was again used to determine the amount of Munc18c and VAMP2 that remained bound following this challenge (lower panel). Data shown are representative of three experiments of this type.

The SM:v-SNARE interaction between Vps45p and Snc2p can be disrupted by the presence of the syntaxin Tlg2p [11]. With this in mind, we performed a series of competition experiments using untagged Sx4 and VAMP2 cytosolic domains (Fig 3). The addition of increasing amounts of VAMP2 had no effect on pre-formed complexes of Sx4:Munc18c (Fig 3A). In contrast, addition of Sx4 (but not the non-cognate syntaxin, Sx16) readily displaced VAMP2 from VAMP2:Munc18c complexes (Fig 3B).

SM proteins clearly play a central role in SNARE-mediated membrane traffic. Here we have focussed on the SM protein, Munc18c, which regulates fusion mediated by the Sx4/SNAP23/VAMP2 complex. This complex regulates the delivery of GLUT4-containing vesicles to the surface of insulin-sensitive cells. In this study, we have shown that Munc18c inhibits membrane fusion catalysed by its cognate SNARE complex. Our findings are consistent with the observation that homozygous knockout of Munc18c in mice results in an increased sensitivity of GLUT4 exocytosis in response to insulin, suggesting that Munc18c negatively regulates GLUT4 exocytosis [20]. In addition, over-production of Munc18c in 3T3-L1 adipocytes has been shown to inhibit insulin-stimulated GLUT4 translocation [21]–[23].

However, others have reported a positive role for Munc18c in the insulin-stimulated delivery of GLUT4 to the plasma membrane of adipocytes [22], [23]. It is important to note that other SM proteins, Munc18a and Sec1p, accelerate fusion catalysed by their cognate SNARE complexes in the liposome fusion assay [12], [16]. Given the structural similarities between SM proteins [3], it seems unlikely that different members of this family perform opposing regulatory functions. Hence, our finding that Munc18c inhibits rather than stimulates membrane fusion may indicate that Munc18c requires a further level of regulation in order to stimulate SNARE-mediated membrane fusion. One possibility is that SM proteins adopt distinct conformations within the cell. Indeed, we have previously characterised a dominant negative version of the yeast endocytic SM protein Vps45p which appears to be locked in a conformation that binds to the assembled SNARE complex [11]. It may be that the recombinant Munc18c used here favours an inactive conformation *in vitro*, whereas the proteins used to demonstrate a stimulatory effect of Munc18a and Sec1p favour an active conformation. It is possible that Munc18c, like other SM proteins [30], is subject to post-translational modifications which may dictate its conformation. Indeed, Munc18c has been shown to be phosphorylated in response to multiple agonists in several cell types [31]–[33]. Intriguingly, phosphorylation of Munc18c has recently been shown to decrease its interaction with Sx4 and promote an interaction with Doc2β [34]. Future research will focus on how such regulatory mechanisms operate on Munc18c to control the insulin-stimulated delivery of GLUT4 to the plasma membrane of adipocytes.

## Materials and Methods

### Plasmids and reagents

Rat GST-tagged Sx4 and GST-VAMP 2 and 3 were from R. Scheller. Human VAMP8 and human VAMP4 were from A. Peden. Anti-Sx4, VAMP2, VAMP4 and VAMP8 and Munc18c were from Synaptic Systems. Lipids were from Avanti Polar Lipids and Triton ×100 and n-octyl-β-**D**-glucopyranoside (OG) were from Sigma. A mutant of VAMP2 lacking the first 30 amino acids was generated by PCR and expressed in pGEX4T-1 as an N-terminal GST-fusion protein. Protein A-tagged Sx16 cytosolic domain was generated by PCR from human syntaxin 16A (obtained from H. Stenmark) [35].

### Purification of SNARE complexes and Munc18c

To purify the syntaxin 4/SNAP23 complex, plasmids containing the entire coding sequence of syntaxin 4 (pQE30) and an N-terminal GST-SNAP23 fusion (pET41a) were co transformed into BL21 DE3 cells and selected on dual antibiotic plates. Colonies were used to start an overnight culture, and the next day a further culture was grown (containing 500 µg/ml ampicillin and 50 µg/ml kanamycin). This overnight culture was then used to inoculate 12 L of Terrific broth containing 200 µg ampicillin and 25 µg kanamycin, and was grown at 37°C with shaking at 250 rpm. An additional 100 µg ampicillin was added each hour. Protein expression was induced by the addition of IPTG to 1 mM when the cells reached an OD600 of approximately 0.6, and incubated overnight at 25°C with shaking at 250 rpm.

The protein complex was purified using glutathione sepharose (Amersham). The cells were broken by two passes through a French press at 950 p.s.i. in buffer A200 (25 mM HEPES pH 7.4, 200 mM KCL, 10% (w/v) glycerol and 2 mM β-mercaptoethanol) containing 4% Triton, complete protease inhibitors (Roche) and 2 mM PMSF. Insoluble matter was removed by centrifugation at 30,000×g for 1 h. The supernatant was incubated with 5 ml of pre-equilibrated glutathione sepharose overnight at 4°C. The beads were washed with 100 ml of A200 containing 1% Triton. The Triton was then exchanged for OG by washing the 10 times with 15 ml of A200 containing 1%OG. The beads were resuspended in an equal volume of A200 containing 1% OG and 125 Units of thrombin were added. The beads were incubated with rotation for 4 h at room temperature, following which the supernatant was collected, aliquoted, snap frozen and stored at −80°C until use.

VAMP2 was expressed as a C-terminally tagged His_6_-myc fusion protein. The culture was grown to an OD600 of 0.8. Protein expression was then induced by the addition of 1 mM IPTG for 3 h at 37°C. Cells were resuspended, broken and the cell lysate centrifuged as outlined for syntaxin 4/SNAP23. The VAMP2 was then purified using Ni-NTA agarose. The beads were then washed once with 100 ml of A200 containing 1% Triton ×100 and 15 mM imidazole. The Triton ×100 was then exchanged for OG by washing the beads 10 times with 10 ml of A200 containing 1% OG and 15 mM imidazole. The protein was eluted from the beads with 3 ml of A200 containing 1% OG and 500 mM imidazole for 30 minutes at 4°C. The supernatant was collected, aliquoted, snap frozen and stored at −80°C until use.

Munc18c was expressed as an N terminal His_6_-fusion protein from the vector pQE30 in M15 cells co-transformed with a vector encoding GroEL. Cells were grown to an OD600 of 0.6 and expression of Munc18c was induced by adding 0.2 mM IPTG overnight at 25°C. Cells were broken by sonication in A400 buffer (25 mM HEPES pH 7.4, 400 mM KCl, 10% (w/v) glycerol, 2 mM β-mercaptoethanol) containing 10 mM Imidazole, EDTA-free complete protease inhibitors and 2 mM PMSF. Insoluble matter was removed by centrifguation. The supernatant was incubated with pre equilibrated Ni-NTA Agarose for 2 h. The beads were washed with 150 ml of A400 containing 15 mM Imidazole. Protein was eluted in A400 buffer with 500 mM imidazole for 1 h then dialysed against A200 without glycerol overnight.

### Reconstitution and in vitro fusion assays

Lipid stocks were prepared in chloroform and stored at −80°C under nitrogen. For t-SNARE liposomes, a 15 mM lipid stock was made up in chloroform containing 85 mol% POPC and 15 mol% DOPS. For v-SNARE liposomes a 3 mM lipid stock was made up in chloroform containing 82 mol% POPC, 15 mol% DOPS, 1.5 mol% NBD-DPPE and 1.5 mol% rhodamine-DPPE [2].

100 µl of 15 mM unlabelled lipid stock, for t-SNARE liposomes, or 500 µl of 3 mM labelled lipid stock, for v-SNARE liposomes was placed at the bottom of a 12×75 mm glass test tube. The chloroform was then evaporated using a stream of nitrogen for 15 min in a fume hood. To ensure that the lipid films were completely dry the samples were then dried for a further 30 min under vacuum. Purified t- or v-SNARE (500 µl) purified as outlined above was then added to each tube. The lipid film was then resuspended, by vortexing for 15 min. After the lipid film was completely resuspended the detergent was diluted below its critical micellar concentration by the addition of 1 ml of Buffer A200 containing 1 mM DTT, drop-wise while the sample was continuously vortexed. To remove any remaining detergent, the samples were placed into pre-equilibrated 3 ml Float-a-Lyzers with a molecular weight cut off of 10,000 and dialysed against 4 L of Buffer A200 containing 1 mM DTT and 4 g of Bio-Beads, with stirring at 4°C overnight. Samples were recovered the following day, and placed at the bottom of a SW60 tube on ice for subsequent separation using gradient centrifugation.

Proteoliposomes were recovered by floatation on a Nycodenz gradient. An equal volume of 80% nycodenz in buffer A200 containing 1 mM DTT was mixed with the recovered dialysate to produce a 40% nycodenz mixture. This was overlaid with 1.5 ml of 30% nycodenz in buffer A200 containing 1 mM DTT. This layer was then overlaid with 250 µl of glycerol free A200 and centrifuged at 65,000×g for 4 h at 4°C. Proteoliposomes were recovered by removal of 400 µl from the top of the gradient, snap frozen and stored at −80°C.

Typically fusion assays were set up by mixing 5 µl of v-SNARE liposome with 45 µl of t-SNARE liposome directly in a well of a 96 well microtitre plate, on ice. This was then sealed and incubated overnight at 4°C. For assays requiring the addition of soluble v-SNARE, 2 µl of purified protein in buffer A200 was added to the t-SNARE liposomes on ice for 10 minutes prior to the addition of v-SNARE liposomes. To correct for the resulting difference in volume 2 µl of A200 was added to all other wells in that run. The fluorescence was measured for 2 h with the excitation set to 460 nm and the emission recorded at 520 nm at 2 min intervals at 37°C. After this period, the plate was removed and 10 µl of 2.5% (w/v) n-dodecylmaltoside was added to each well. The plate was gently mixed for 2 min and then fluorescence was recorded for 40 min at 2 min intervals.

### Pull-Down Assays

5 µg of GST-tagged proteins (GST alone, N-terminal tagged Sx4, VAMP2, VAMP4 and VAMP8), were incubated with 10 µl of glutathione sepharose in binding buffer (150 mM Potassium acetate, 1 mM MgCl_2_, 0.05% Tween 20, 20 mM HEPES pH 7.4) in 200 µl for 1.5 h at 4°C with rotation. Unbound protein was removed by 3 washes with 1 ml binding buffer. 5 µg of HIS-tagged Munc18c protein and binding buffer was added to each tube (final volume 500 µl) and incubated at 4°C overnight with rotation. 20 µl was removed in order to examine protein input. Unbound protein was removed by 3 washes with 1 ml binding buffer plus 0.2% fish skin gelatin (Sigma), followed by 3 washes with 1 ml of binding buffer plus 5% (w/v) glycerol and 4 washes with 1 ml of binding buffer alone. After the final wash, all remaining supernatant was removed and 15 µl of 1× SDS-PAGE sample buffer (with 20 mM DTT) was added to and samples boiled for 5 min. After centrifugation at 14,000×g for 5 min, the supernatant was removed and analysed by SDS-PAGE and/or immunoblotting as outlined in [36]. For the experiments shown in Fig 3, the GST moiety was cleaved using thrombin as described [16].
